# Long-term observation after transplantation of cultured human corneal endothelial cells for corneal endothelial dysfunction

**DOI:** 10.1186/s13287-022-02889-x

**Published:** 2022-06-03

**Authors:** Peng Sun, Lin Shen, Yuan-Bin Li, Li-Qun Du, Xin-Yi Wu

**Affiliations:** 1 Department of Ophthalmology, The Affiliated Yantai Yuhuangding Hospital of Qingdao University, Yantai, China; 2 Department of Ophthalmology, Qilu Hospital of Shandong University, Jinan, China

**Keywords:** Corneal endothelial cell, Orbital adipose-derived stem cell, Conditioned medium, Regenerative medicine, Animal model, Corneal endothelial dysfunction, Cell transplantation, Transcriptome sequencing

## Abstract

**Background:**

Corneal transplantation is the only way to treat serious corneal diseases caused by corneal endothelial dysfunction. However, the shortage of donor corneal tissues and human corneal endothelial cells (HCECs) remains a worldwide challenge. We cultivated HCECs by the use of a conditioned medium from orbital adipose-derived stem cells (OASC-CM) in vitro. Then the HCECs were used to treat animal corneal endothelial dysfunction models via cell transplantation. The purpose of this study was to conduct a long-term observation and evaluation after cell transplantation.

**Methods:**

Orbital adipose-derived stem cells (OASCs) were isolated to prepare the conditioned medium (CM). HCECs were cultivated and expanded by the usage of the CM (CM-HCECs). Then, related corneal endothelial cell (CEC) markers were analyzed by immunofluorescence. The cell proliferation ability was also tested. CM-HCECs were then transplanted into monkey corneal endothelial dysfunction models by injection. We carried out a 24-month postoperative preclinical observation and verified the long-term effect by histological examination and transcriptome sequencing.

**Results:**

CM-HCECs strongly expressed CEC-related markers and maintained polygonal cell morphology even after 10 passages. At 24 months after cell transplantation, there was a CEC density of more than 2400 cells per square millimeter (range, 2408–2685) in the experimental group. A corneal thickness (CT) of less than 550 μm (range, 490–510) was attained. Gene sequencing showed that the gene expression pattern of CM-HCECs was similar to that of transplanted cells and HCECs.

**Conclusions:**

Transplantation of CM-HCECs into monkey corneal endothelial dysfunction models resulted in a transparent cornea after 24 months. This research provided a promising prospect of cell-based therapy for corneal endothelial diseases.

**Supplementary Information:**

The online version contains supplementary material available at 10.1186/s13287-022-02889-x.

## Background

The human corneal endothelium (CE) consists of a monolayer of hexagonal corneal endothelial cells (CECs) lining the posterior surface of the cornea and corneal transparency is maintained via pump and barrier functions [1]. Multiple factors such as Fuchs endothelial corneal dystrophies (FECD), Peter’s anomaly, Iridocorneal endothelial syndrome (ICE), intraocular surgery, hypoxia, infection, or trauma can damage human corneal endothelial cells (HCECs), which can lead to corneal endothelial damage and loss of CECs [2–7]. This pathological state can be compensated for by the natural spread of the remaining CECs in healthy persons [8]. However, CECs have limited proliferative capacity in vivo [9]. When the CEC density diminishes to a critical value (< 500 cells/mm^2^), corneal endothelial dysfunction occurs. Corneal decompensation and abnormal corneal hydration will follow, resulting in corneal thickening and haziness. This condition can ultimately cause severe impairment of vision [2, 3, 10, 11].

The current treatments for corneal endothelial dysfunction include traditional penetrating keratoplasty (PKP), Descemet’s stripping endothelial keratoplasty (DSEK), Descemet's stripping automated endothelial keratoplasty (DSAEK), and Descemet’s membrane endothelial keratoplasty (DMEK), all of which rely on a donor cornea with healthy HCECs [12–15]. There are only about 185,000 corneal transplants performed worldwide per year due to a serious shortage of donor corneas which cannot satisfy the demand of clinical practice [16]. New treatments that do not depend on donor tissue therefore need to be devised. Novel therapeutic modalities such as cell-based therapy and regenerative medicine show good prospects [17–20]. In a previous study we innovatively cultivated HCECs with conditioned medium (CM) obtained from human orbital adipose-derived stem cells (OASCs). The CM-HCECs (HCECs cultured with conditioned medium) obtained a good proliferative ability. Then we produced rabbit and monkey animal corneal endothelial dysfunction models and treated them via cell transplantation. During the observation of 10 months after cell transplantation, the opaque and edematous corneas rapidly recovered and maintained normal thickness and transparency [21].

In the present work, we conducted a 24-month preclinical observation and examinations after CM-HCECs transplantation into monkey endothelial dysfunction models. Further transcriptome sequencing was carried out on HCECs, CM-HCECs, and transplanted cells. The results showed that the treated corneas remained transparent and had moderate thickness. The endothelial dysfunction models were successfully treated. The transplanted cells displayed good therapeutic ability and adapted well to the microenvironment of the host. The study indicated that more useful cells would be available for research and clinical cell-based therapy for corneal endothelial dysfunction in the future.

## Methods

### Cell culture and evaluation of OASCs and HCECs

Conditioned medium obtained from human orbital adipose-derived stem cells (OASC-CM) and HCECs were prepared as previously described. Orbital adipose tissues were collected from 15 patients aged between 23 and 65 (45.3 ± 9.8) following blepharoplastic surgeries. Briefly, OASCs were isolated and cultured in DMEM-LG (Hyclone, Logan, USA) supplemented with 10% FBS (Gibco, Grand Island NY, USA) and 10% penicillin–streptomycin (Sigma, Darmstadt Merck, Germany), and incubated at 37 °C in 5% CO_2_ [22, 23]. Related cell markers of OASCs were analyzed by flow cytometry and vimentin was detected by immunofluorescence. OASCs were washed three times with phosphate-buffered saline (PBS) when they were at 60–80% confluence, and the medium was replaced with basal growth medium. The OASCs were kept for an additional 12–24 h. The medium was then collected and filtered (0.22 μm) and stored at − 80 °C.

HCECs were obtained from discarded corneal-scleral rings after penetrating keratoplasty (PK) and from the Eye Tissue Bank of Shandong Province, China. The age of donors ranged from 15 to 78 (*n* = 10). The endothelial layers of two donors were directly stripped without any treatment and used for RNA sequencing. These two HCECs were marked as HCEC 1 and HCEC 2. CM-HCECs were generated from eight other donors. Briefly, the Descemet’s membranes (DM) containing HCECs were stripped and incubated in basal culture medium (BM) for stabilization, followed by digestion with 1 mg/ml collagenase type I (Sigma, Darmstadt Merck, Germany) [24]. The BM was composed of Opti-MEM-I (Gibco, Carlsbad, USA), 8% FBS, 5 ng/ml human epidermal growth factor (hEGF; PeproTech, Cranbury NJ, USA), 20 μg/ml ascorbic acid (Sigma, Darmstadt Merck, Germany), 200 mg/L calcium chloride, 0.08% chondroitin sulfate, and 50 μg/ml penicillin–streptomycin (Solarbio, Beijing, China) [25]. After digestion the HCECs were cultured in the BM containing 10% OASC-CM as CM-HCECs. CEC-related functional proteins Na^+^/K^+^ ATPase and tight junction protein zona occludens 1 (ZO-1) were evaluated by immunofluorescence according to the previous protocol [21]. Cell proliferation, migration, and repair capacity were detected with a Cell Counting Kit-8 (CCK-8; Bestbio, Nanjing, China) Assay and Wound Healing Assay according to the manufacturer’s protocol and our previous method [21]. Passage 9 (P9) and passage11 (P11) of CM-HCECs were used for cell transplantation in the study.

### Animals

Four rhesus monkeys weighing 3.0–4.0 kg (3 to 5 years of age; HongLi Medical Animal Experimental Research Center, Jinan, Shandong Province, China) were used for animal experiments. All animals were treated in accordance with the Institutional Animal Care guideline of Hongli Medical Animal Experimental Research Center, Shandong Province, China, and approved by their Association for Laboratory Animal Care (approval number 2016-0803).

### Transplantation of CM-HCECs into the monkey corneal endothelial dysfunction models

**Fig. 1:**
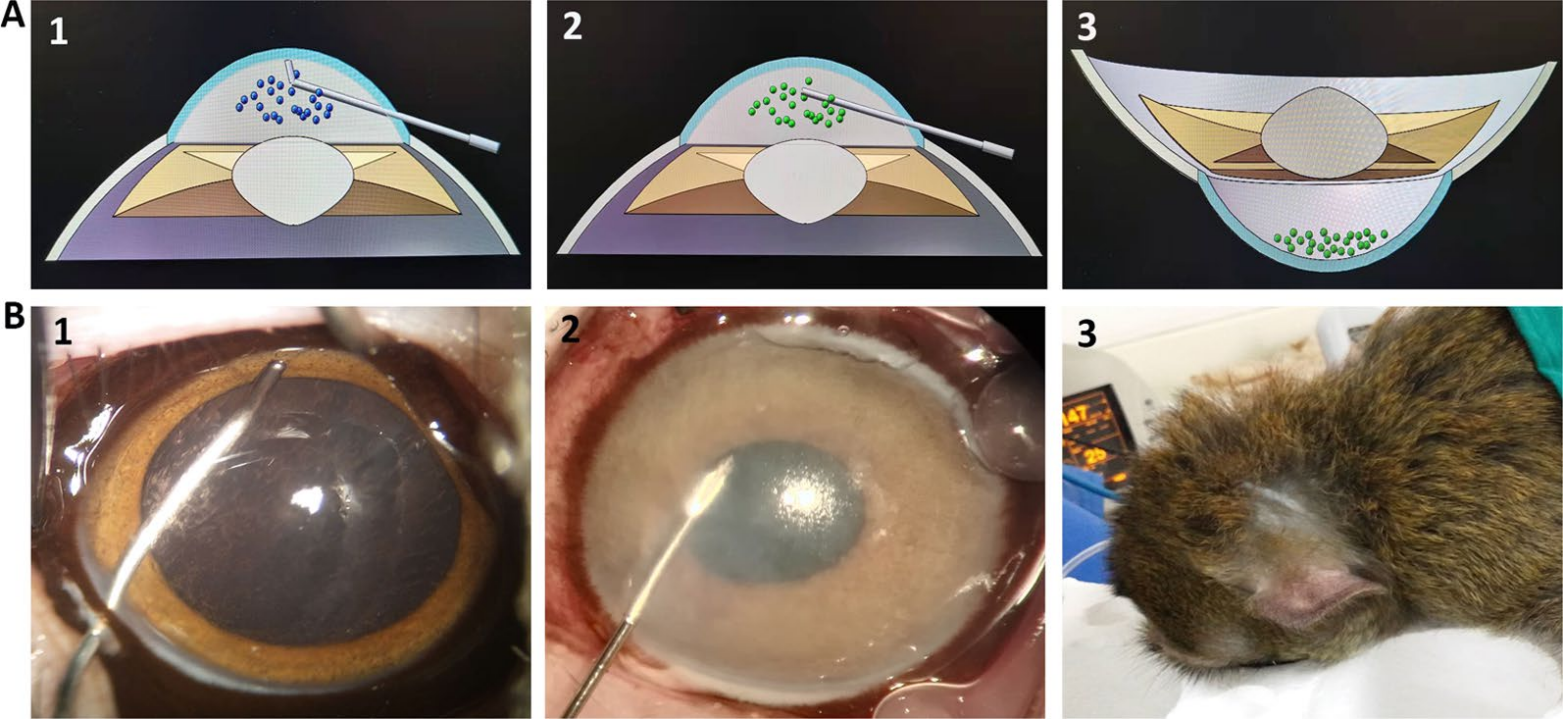
Schema of CM-HCECs transplantation into the monkey corneal endothelial dysfunction model. **A1** Scraping of CECs (the blue dots) on the DM. **A2** Injection of CM-HCECs (the green dots) into the anterior chamber. **A3** The monkey’s eyes were kept in a face-down position for the CM-HCECs (the green dots) to sink onto the DM. **B1** The CECs were completely scraped from the DM of the monkey model with a modified irrigator needle. **B2** CM-HCECs suspended in MEM were injected into the anterior chamber with an insulin needle. **B3** The monkeys were kept in the face-down position immediately after cell transplantation

Monkeys were randomly divided into the experimental group (*n* = 3) and the control group (*n* = 1). The nonsurgical eyes of monkeys were used as the normal group. Monkey corneal endothelial dysfunction models and CM-HCECs transplantation were performed according to our previous method [21, 26, 27]. Briefly, monkeys were under general anesthesia of ketamine hydrochloride. The CECs were mechanically scraped with a modified irrigator needle (WEGO, Weihai, China) from the DM of the four monkeys (Fig. 1A1, B1). The aqueous humor (50 μl) was first extracted from the anterior chamber. CM-HCECs (2.7 × 10^5^ cells for each eye) were suspended in 50 μl culture medium and injected into the anterior chamber of three monkeys as the experimental group (Fig. 1A2, B2). The other monkey had only 50 μl culture medium injected without any cells as the control group. A peribulbar injection of triamcinolone and a sub-conjuctival injection of dexamethasone were given at the end of the surgery. All the monkeys were then immediately kept in a face-down position for 5 h under general anesthesia (Fig. 1A3, B3). Tobramycin and Dexamethasone drops were given 4 times a day. The corneas were examined by a slit-lamp microscope, AccuPen Handheld Tonometer (Accutome), OCT (Carl Zeiss), non-contact specular microscope (Topcon), gonioscope (Volk), B-ultrasonography (Suoer), and a fundus camera (Carl Zeiss) at certain times after surgery. Three monkeys in the experimental group were euthanized at 24 months after the transplantation and were labeled as the transplantation after 2 years (TR2Y) group. The monkey of the control group was also euthanized 24 months after surgery. Eyes were postoperatively removed. The corneas were divided into several parts, one for RNA-Sequencing, and one for immunofluorescent staining of frozen sections, and the other part was subjected to hematoxylin and eosin (H&E) staining.

### Immunofluorescence and histological examination

After eyes were postoperatively removed part of the cornea was embedded in Tissue-Tek optimum cutting temperature compound (Sakura, Torrance, USA) and sectioned into 5 μm slices. The frozen slices were subjected to standard immunofluorescent staining [21]. Primary antibodies were anti-Na^+^/K^+^ ATPase *α*-1 (1:200, Millipore, Darmstadt, Germany) and anti-Zo-1 (1:100, Santa Cruz, Dallas, USA). Part of the cornea was fixed in 4% formaldehyde and subjected to standard H&E staining.

### RNA-sequencing

The total RNA of HCECs, CM-HCECs, and corneal endothelial cells of TR2Y groups were isolated using TRIzol reagent (Life Technologies, Carlsbad, California, USA) according to the manufacturer’s protocol. RNA-seq transcriptome library was prepared following the TruSeqTM RNA sample preparation Kit from Illumina (San Diego, CA, USA) using 1 mg of total RNA. Sequencing was performed by Majorbio Biotech (Shanghai, China) using the Illumina HiSeq 4000 150 bp Paired-End Platform. The raw paired end reads were trimmed and quality controlled by SeqPrepand Sickle with default parameters. Then clean reads were separately aligned to the human reference genome with orientation mode using HIASAT software. The mapped reads of each sample were assembled by StringTie. The HCEC RNA-seq data in this study represented the average value of HCEC 1 and HCEC 2. The state of CM-HCECs from different donors were consistent after culture and passages so that one of the CM-HCECs was selected for RNA sequencing. HCECs and CM-HCECs came from different individuals of the same species. RNA sequencing was used to compare the expression levels between the different samples.

To identify differential expression genes (DEGs) between two different samples, the expression level was calculated according to the TPM method. RSEM was used to quantify gene abundances. The R statistical package software EdgeR was utilized for differential expression analysis. Genes with FDR < 0.05 and |log2FC|≥ 1 were considered as significant. Venn analysis (TPM > 1) was used to demonstrate common and uniquely expressed gene transcripts between samples. The correlation analysis provided a basic reference for the analysis of differentially expressed genes. GO functional enrichment analysis was performed by Goatools in order to identify which DEGs were significantly enriched at Bonferroni-corrected *P*-value ≤ 0.05 compared with the whole transcriptome background.

### Statistical analysis

All the data were presented as the mean ± SEM. Experiments with two treatments and/or conditions were analyzed using a two-tailed Student’s t test by the SPSS v24.0 software. Image processing was performed using GraphPad Prism 8.0.2 software and Adobe Photoshop CC 20.0.5 software. *P* < 0.05 was considered statistically significant.

## Results

### Characterization of OASCs and CM-HCECs

#### Promoted proliferation ability of HCECs by OASC-CM

In the study human OASCs were plastic adherent, spindle-shaped, fibroblast-like cells. They highly expressed vimentin by immunofluorescence and CD29, CD105, CD49e, and CD166 determined by flow cytometry, suggesting their endothelial and stem cell origins (Additional file 1: Fig. S1A–D).

**Fig. 2:**
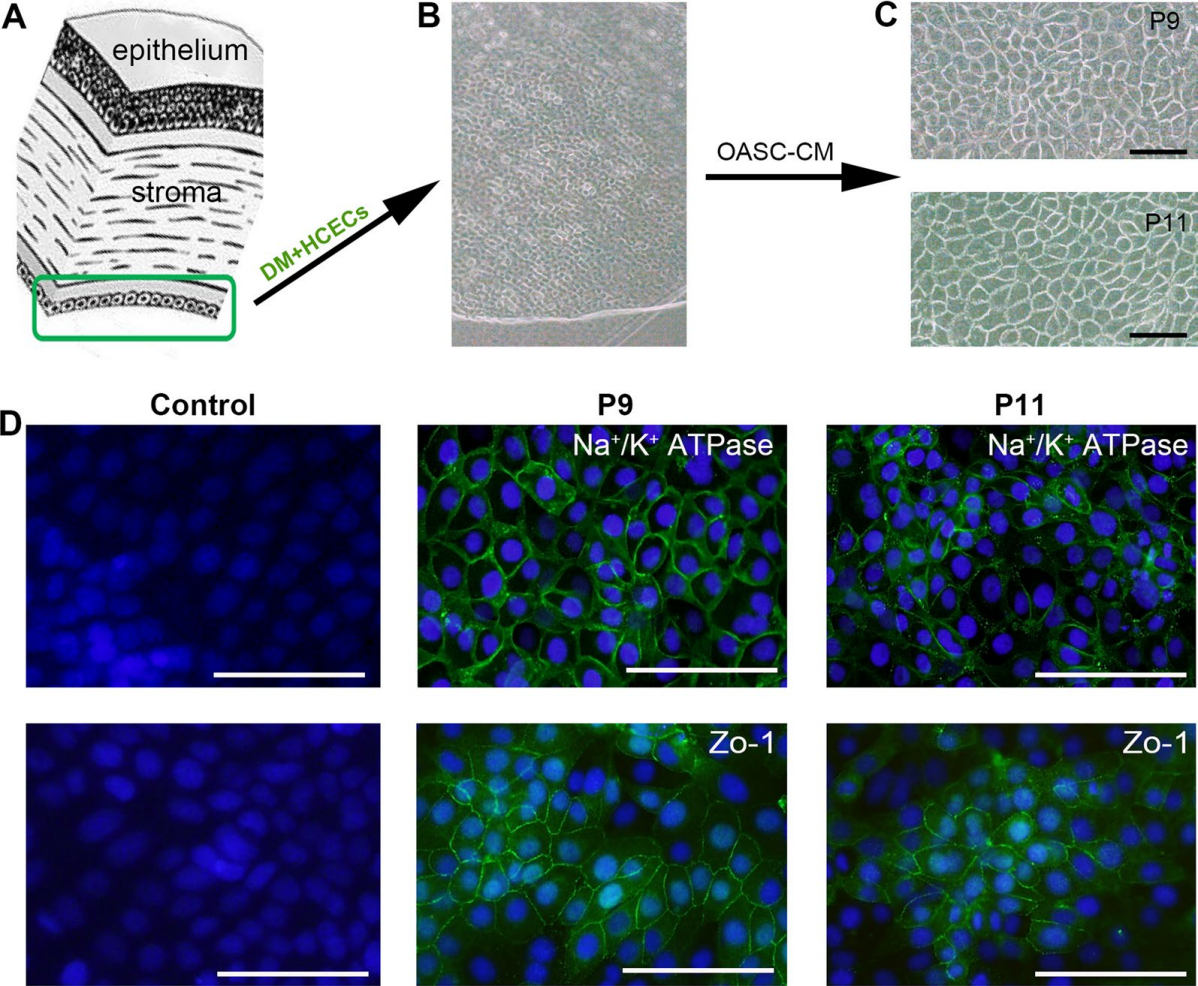
Isolation of HCECs and cell culture, characteristics of CM-HCECs. **A, B** Peeled DM layer that contained CECs. **C** The morphology of different passages of CM-HCECs (P9, P11). **D** Expression of CEC relative markers by immunofluorescence. Scale bar: 100 μm

HCECs displayed typical hexagonal morphology on the DM in vivo (Fig. 2A, B). When cultured in the BM, HCECs had a fibroblastic change, became larger with vacuoles, and showed an endothelial-to-mesenchymal transition (EMT) after 4–5 passages (Additional file 1: Fig. S1E). However, when cultured in OASC-CM, HCECs maintained a polygonal morphology and contact-inhibited monolayer even after 10 passages (Fig. 2C and Additional file 1: Fig. S1F). Also, CM-HCECs expressed CEC-related functional proteins such as Na^+^/K^+^ ATPase and ZO-1 (Fig. 2D).

### Long-term observation after transplantation of cultivated CM-HCECs into the primate models

#### Restoration of corneal endothelial function

**Fig. 3:**
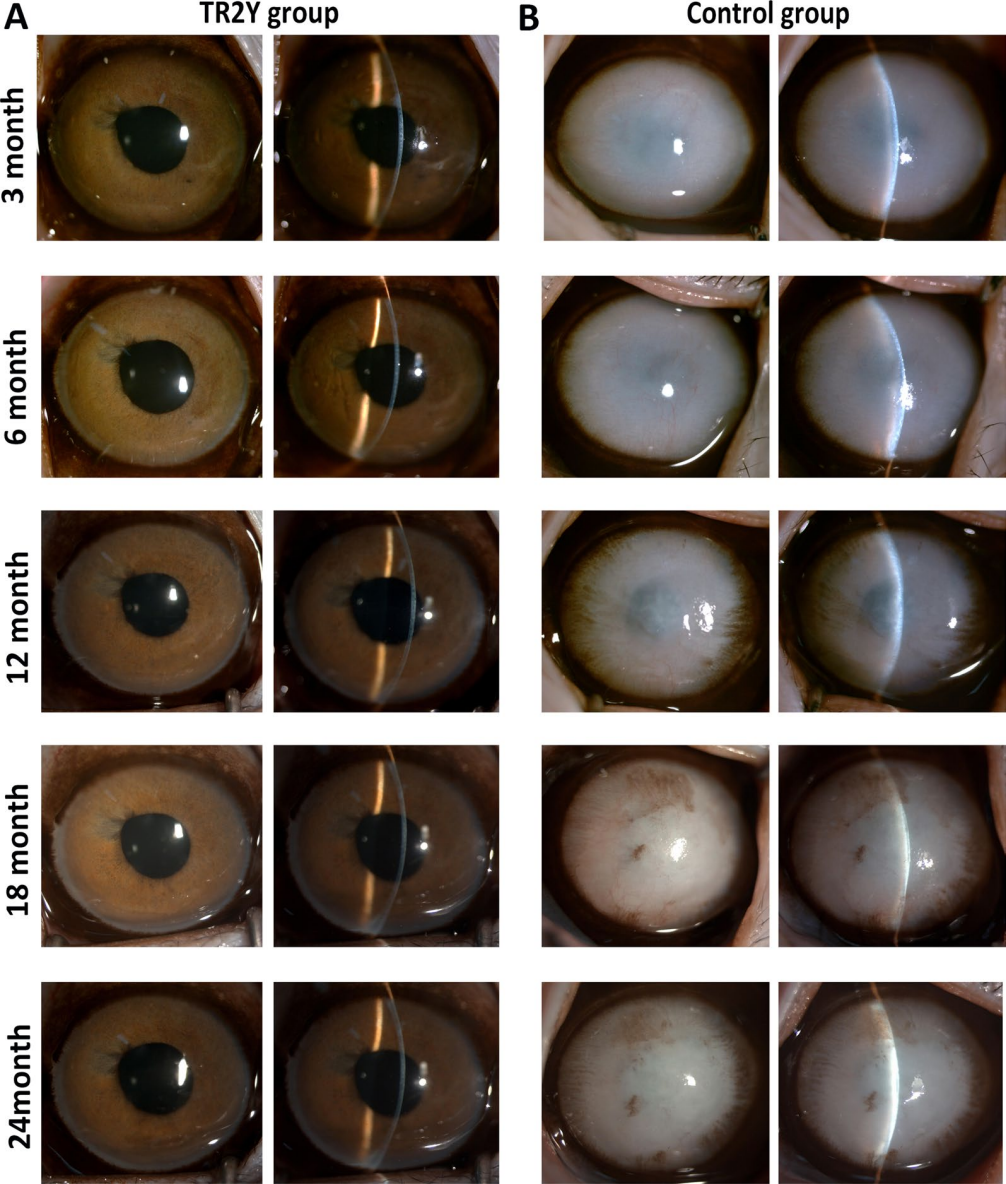
Observation of the TR2Y group and the control group during 24 months after transplantation of CM-HCECs. **A** Corneal transparency, thickness, and anterior chamber condition were examined by slit-lamp in the TR2Y group (monkey 1). **B** Corneal transparency, thickness, and anterior chamber condition were examined by slit-lamp in the control group

Slit-lamp microscopy and OCT showed that the corneas recovered transparency at about 7 days after cell transplantation in the TR2Y group. Keratic precipitates (KP), anterior chamber exudation at 10–14 days, and posterior synechiae of the pupil at 21–28 days could be noticed after the transplantation. KP and exudation significantly decreased about 1 month after cell transplantation. Posterior synechiae of the pupil returned to normal at about 2 months after operation (Additional file 2: Fig. S2). The depth of the anterior chamber was normal, the iris texture was clear, the pupil was round with normal direct light reflection, and the crystalline lens was transparent from 3–24 months. The corneas of the TR2Y group remained transparent at 24 months after surgery (Fig. 3A), whereas the cornea in the control group had obvious corneal opacity and stroma edema. The iris, pupil, and crystalline lens could not be seen at 3 months after surgery. Also, obvious corneal neovascularization was detected by the slit lamp microscope (Fig. 3B).

**Fig. 4:**
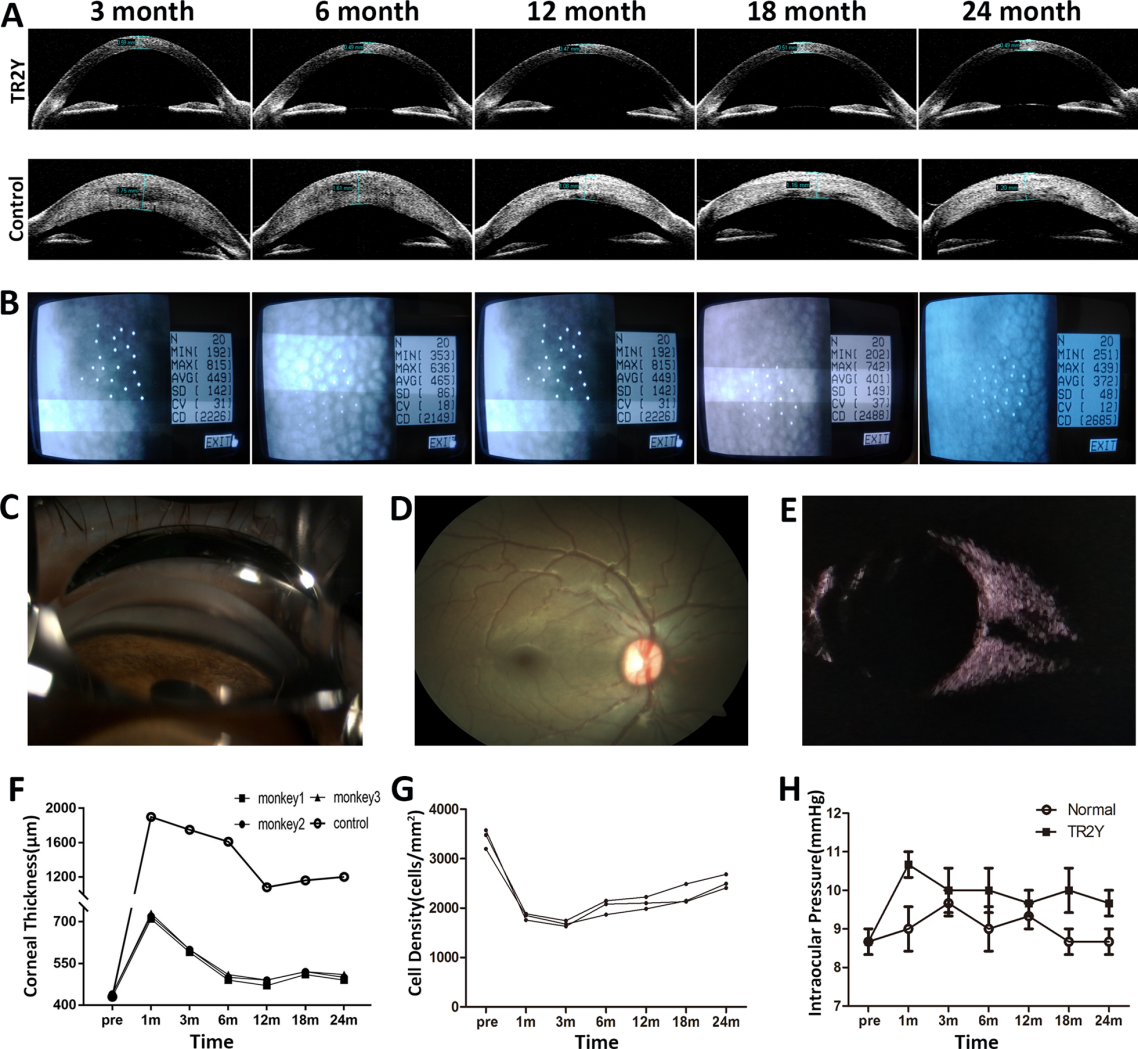
Ophthalmologic examination results of the TR2Y group and the control group after cell transplantation. **A** The central corneal thickness (CCT) in the TR2Y group and the control group by OCT. **B** Cells were detected by noncontact specular microscopy in the TR2Y group. **C**–**E** Images of anterior chamber angle, fundus, and ocular B-mode ultrasound in the TR2Y group. **F** CCT was measured by OCT in the TR2Y group (monkey 1, 2, 3) and the control group before and after surgery. **G** Changes of cell density in the TR2Y group (monkey 1, 2, 3). **H** IOP was measured in the TR2Y group and the normal group during the 24 months observation

The central corneal thickness (CCT) of the TR2Y group was about 720 μm at 1 month after surgery. After that, the CCT was between 600 and 700 μm at 1–3 months. It was stable at about 500 μm from 6 to 24 months after surgery. Yet the CCT of the control group reached 1900 μm at 1 month after surgery and remained above 1000 μm during the next 23 months (Fig. 4A, F). The CECs in the TR2Y group showed multilateral morphology under the examination of a non-contact specular microscope. The average endothelial cell density (CD) was about 1800 cells/mm^2^ at the first month after surgery. It decreased to about 1682 cells/mm^2^ at the third month. After that, the number of CECs continuously increased. The average CD reached 2530 cells/mm^2^ at the end of 24 months (Fig. 4B, G). The CD of CECs in the control group could not be detected with specular microscopy or a confocal microscope because of the obvious corneal opacity and edema (Fig. 3B). In addition, a gonioscope, fundus photography, and B-mode ultrasound showed no pathological changes of eyes in the TR2Y group (Fig. 4C–E). There was no abnormal increase of intraocular pressure (IOP) in the TR2Y group during the 24-month period of evaluation (Fig. 4H).

### Histological examination and Immunofluorescence

#### Formation of a monolayer on the DM by functional cells

**Fig. 5:**
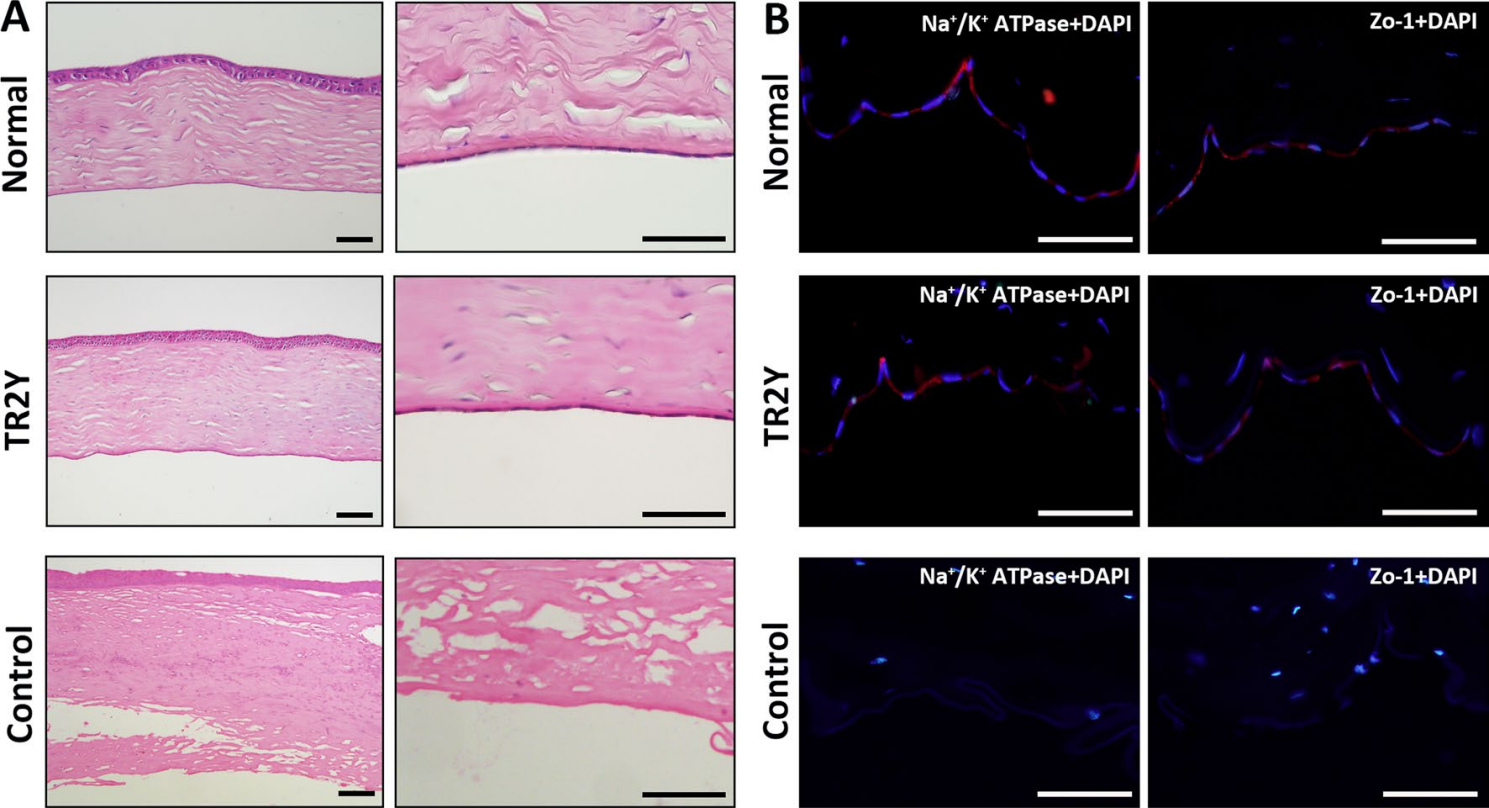
Histological examination and immunofluorescence of the normal group, the TR2Y group, and the control group. **A** H&E staining of corneas in the three groups. **B** Immunofluorescent staining of Na^+^ /K^+^ ATPase and Zo-1 in the three groups (blue: DAPI, red: Na^+^ /K^+^ ATPase, Zo-1). Scale bar: 100 μm

HE staining showed that the corneal thickness of the TR2Y group was similar to that of the normal group. Cells created a closely arranged monolayer on the DM. The cornea of the control group was significantly thickened, and almost no cells were detected on the endothelial surface. There were more inflammatory cells in the corneal stroma, irregular arrangement and fracture of collagen fibers, and vacuolar changes in the corneal epithelium (Fig. 5A).

Immunofluorescent staining of frozen sections showed that Na^+^/K^+^ ATPase and ZO-1 were strongly expressed in the corneal endothelium of the TR2Y group and the normal group, suggesting the pump function and tight junction function of CECs. On the contrary, there was almost no nuclear staining on the DM in the control group. The staining results of Na^+^/K^+^ ATPase and ZO-1 were negative (Fig. 5B).

### The differentially expressed genes and correlation in HCECs, CM-HCECs, and TR2Y cells

**Fig. 6:**
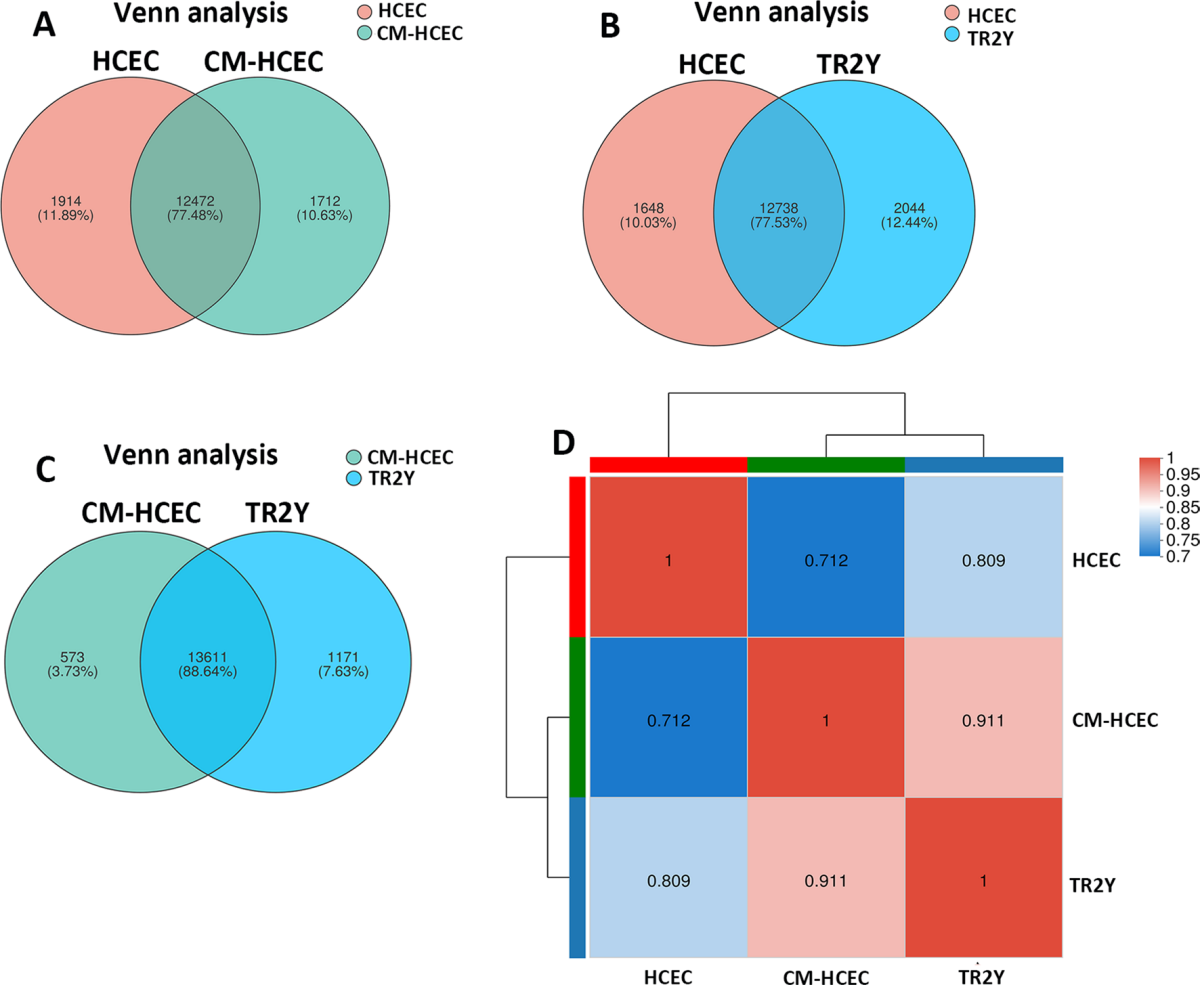
Correlation of gene expression between HCECs, CM-HCECs, and TR2Y cells. **A** Venn analysis of HCECs and CM-HCECs. **B** Venn analysis of HCECs and TR2Y cells. **C** Venn analysis of CM-HCECs and TR2Y cells. **D** The correlation analysis of HCECs, CM-HCECs, and TR2Y cells.

RNA-seq was carried out to study the gene expression of HCECs, CM-HCECs, and cells after transplantation (TR2Y cells). The result of Venn analysis displayed the co-expressed genes and specially expressed genes between samples. The Venn analysis of HCECs and CM-HCECs showed that 12,472 genes were co-expressed, accounting for 77.48%. The number of specific genes of HCECs and CM-HCECs was 1914 and 1712, accounting for 11.89% and 10.63%, respectively (Fig. 6A). The Venn analysis of HCECs and TR2Y cells showed that 12,738 genes were co-expressed, accounting for 77.53%. The number of specific genes of HCECs and TR2Y cells was 1648 and 2044, accounting for 10.03% and 12.44%, respectively (Fig. 6B). The Venn analysis of CM-HCECs and TR2Y cells showed that 13,611 genes were co-expressed, accounting for 88.64%. The number of specific genes of CM-HCECs and TR2Y cells was 573 and 1171, accounting for 3.73% and 7.63%, respectively (Fig. 6C).

Correlation analysis was carried out to detect the relativity between samples. The r value between HCECs and CM-HCECs was 0.712. The r value between HCECs and TR2Y cells was 0.809. The r value between CM-HCECs and TR2Y cells was 0.911. (Fig. 6D).

### Differential expression gene analysis of CM-HCECs and TR2Y cells

**Fig. 7:**
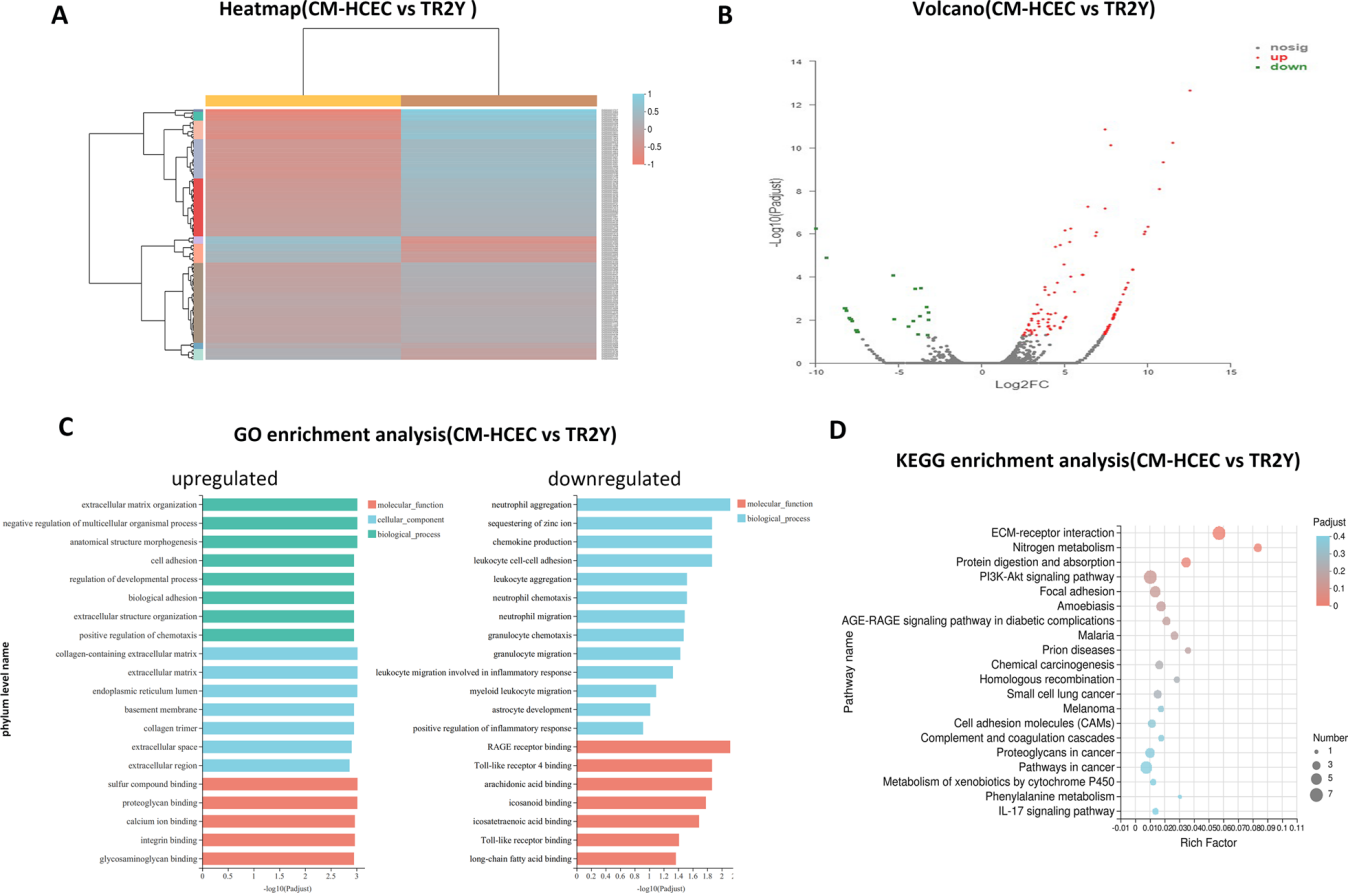
Differential expression gene analysis of CM-HCECs and TR2Y cells. **A** Heatmap of CM-HCECs versus TR2Y cells. **B** Volcano of CM-HCECs versus TR2Y cells. **C** GO enrichment analysis of CM-HCECs versus TR2Y cells. The up-regulated genes (left) and the down-regulated genes (right). **D** KEGG enrichment analysis of CM-HCECs versus TR2Y cells

Expression variance analysis was carried out to detect differential expression genes (DEGs) between CM-HCECs and TR2Y cells. 129 significant DEGs were detected between CM-HCECs and TR2Y cells, among which 104 genes (80.6%) were up-regulated and 25 genes (19.4%) were downregulated (Fig. 7A, B).

The GO enrichment analysis of CM-HCECs and TR2Y cells showed the top 20 GO sets of abundance included classification of biological processes, cellular components, and molecular function. The results showed that the up-regulated genes were mainly enriched in function of cell adhesion, the extracellular region, and the different binding region. The down-regulated genes were mainly enriched in function of inflammation (Fig. 7C). The KEGG enrichment analysis of CM-HCECs and TR2Y cells showed the top 20 KEGG sets of abundance. ECM-receptor interaction, nitrogen metabolism, protein digestion and absorption, the PI3K-Akt signaling pathway and focal adhesion were the top 5 sets in abundance (Fig. 7D). The up-regulated DEGs were mainly enriched in the extracellular matrix (ECM) of these pathways such as collagen, laminin, and fibronectin.

## Discussion

In recent years corneal endothelial keratoplasty has brought new technologies for the treatment of corneal endothelial dysfunction [28–30]. But these methods have disadvantages such as technical difficulty, cell loss, lamellae dislocation, graft rejections, and graft failure after surgery [31–34]. More importantly, these operations cannot be performed without donor corneas, especially healthy HCECs. The rapid development of regenerative medicine provided a solution for obtaining available HCECs. For example, Lin, S et al. studied the trans-differentiation of skin-derived precursors (SKPs) into HCEC-like cells and achieved the preliminary results in the treatment of animal corneal endothelial dysfunction [35]. Other researchers studied the effect of pharmacological agents on CEC proliferation, such as rho-associated protein kinase inhibitor Y-27632 and inhibitors of the p38 mitogen-activated protein kinase (MAPK) [17, 36]. Kinoshita, S et al. used cell injection with Y-27632 to treat human bullous keratopathy and the experimental results were gratifying [37]. These cells had some functions of HCEC. However, the common disadvantage of the cells is that they cannot be subcultured over several passages and amplified in large numbers. So a sufficient number of HCECs could not be obtained for basic research and clinical treatment.

The worldwide shortage of donor corneas and HCECs is expected to be completely solved with the rapid development of tissue engineering and regenerative medicine [20, 38, 39]. In our previous study, we prepared a conditioned medium obtained from OASCs (OASC-CM) and cultivated HCECs with OASC-CM (CM-HCECs). The results of in vitro experiments showed that CM-HCECs can highly express CEC related markers (N-Cadherin, Na^+^/K^+^ ATPase, and ZO-1) and maintain the characteristic polygonal cell morphology even after 10 passages. In order to test the therapeutic ability of CM-HCECs in vivo, we carried out animal experiments of corneal endothelial dysfunction in rabbit and monkey models. The results of 10 months observation after surgery showed that the corneas can recover and remain transparent for a short time which preliminarily proved the therapeutic effect of CM-HCECs [21].

In the present study, we conducted monkey experiments and observed 24 months after cell transplantation aiming to obtain long-term data. Corneal thickness and endothelial cell density are two key indicators in evaluating the recovery of corneal function. Through the experimental results we discovered that destruction of CECs, loss of pump and barrier functions, and activated stromal cells caused corneal edema. The corneal thickness of the control group reached a maximum of 1900 μm at 1 month after the operation. This was similar to the results of Wu et al. [40]. In the TR2Y group, the average CCT was 720 μm, which was significantly lower than that in the control group, indicating that the transplanted cells worked. The CT gradually decreased with the recovery of corneal endothelial function in the TR2Y group during 1–6 months after cell transplantation. The CCT remained at about 500 μm 6–24 months after surgery and the corneas were transparent, whereas, in the control group the CT was continuously over 1000 μm and the cornea was opaque. The CD of CECs in the control group could not be detected. In the TR2Y group, the average endothelial cell density was the lowest (1682 cells/mm^2^) in the third month, which was considered to be related to postoperative immune reaction, microenvironment change, apoptosis caused by inflammatory factors, and death by cell senescence [41, 42]. After 3 months, the cell density gradually increased, indicating that the transplanted cells had adapted to the new microenvironment and remained stable. At 24 months after cell transplantation, the average CD in the TR2Y group remained at above 2400 cells/mm^2^, which was slightly lower than that of over 3000 cells/mm^2^ in normal monkeys before the operation. The CD of CECs in normal adults is above 2000 cells/mm^2^. In addition, the corneas of the TR2Y group remained transparent. Therefore, the CD in our study conformed to the normal physiological state. Also, there were no pathological changes in eyes of the TR2Y group as determined by a gonioscope, fundus photography, and B-mode ultrasound. The result indicated that transplanted cells harmoniously coexisted with the host. Histological examination and immunofluorescence also showed that the transplanted cells formed a single cell layer on the DM and had tight junctions and pump function.

RNA-sequencing was used to study the gene expression pattern of HCECs, CM-HCECs, and TR2Y cells after cell transplantation. Venn analysis and correlation analysis showed that the proportion of co-expressed genes and the correlation coefficient in HCECs, CM-HCECs, and TR2Y cells were quite high. The results of GO annotation analyses also showed that the three cell types were similar in gene expression pattern and belonged to the same type of cells.

Venn analysis showed that the CM-HCECs and TR2Y cells had a high proportion of co-expressed genes. Correlation analysis showed that there was a high correlation between CM-HCECs and TR2Y cells and the r value was 0.911. It showed that most cells maintained their original expression pattern and remained in a stable state after the transplantation. Venn analysis showed that the proportion of co-expressed genes of HCECs and TR2Y was 77.53% which was higher than that of HCECs and CM-HCECs (77.48%). The correlation analysis showed that the r value was 0.809 in HCECs vs TR2Y cells and 0.712 in HCECs vs CM-HCECs. The result indicated that the transplanted cells were closer to HCECs. It could be seen from the analysis of the expression difference between CM-HCECs and TR2Y groups that transplanted cells were obviously enhanced in the extracellular matrix, cell adhesion, and other related functions. Meanwhile, inflammation-related functions are inhibited. We therefore consider that the CM and the microenvironment of monkey anterior chamber together make the transplanted cells survive and work [26].

In this study, KP, anterior chamber exudation, and posterior synechiae of the pupil occurred after the transplantation. This may due to the immune reaction caused by some transplanted cells falling onto the lens or into the anterior chamber. In addition, heterologous grafts and usage of serum containing medium could also cause immune or rejection reactions [21, 43]. But the reactions were moderate and could be controlled by conventional therapy. This may benefit because the cultured HCECs are endowed with the function of immunomodulation by the CM [44, 45].

Cell transplantation by injection has the advantages of a small incision and simple operation. On the other hand, this method also has limitations such as inaccurate location of cells and the requirement for a special body position after surgery.

In this preclinical research, the general condition of the monkeys was good during 24 months of postoperative observation. There were no indications of abnormal intraocular pressure, lens opacity, and changes of the fundus. Certainly, there are many unknowns to explore, such as the underlying mechanisms of improving the proliferation ability and suppressed EMT, etc. We have started relevant research, and in the future we will improve the experimental method and hope to conduct clinical trials.

## Conclusions

We innovatively cultivated HCECs with OASC-CM and first transplanted the CM-HCECs into the monkey corneal endothelial dysfunction models. In the long-term observation after cell transplantation, the corneas of the experimental group maintained transparency and appropriate CT. The transplanted cells successfully treated the monkey corneal endothelial dysfunction and remained stable in the host. Then we acquired a further understanding of the cells in terms of genes and proteins. The gene expression pattern of transplanted cells was close to that of HCECs. These results add to the field of cell therapy by confirming the safety and potential clinical advantages for corneal endothelial dysfunction. Our research provides more cell resources and ideas for regenerative medicine and tissue engineering.

## Supplementary Information

**Additional file 1: Figure S1.** Cell culture, and characteristics of human orbital adipose-derived stem cells (OASCs), HCECs (cultivated in BM), and CM-HCECs. (A), (B) OASCs were adherent, spindle-shaped, fibroblast-like cells under phase-contrast microscopy. (C) Expression of vimentin as determined by immunofluorescence. (D) Expression of related cell markers determined by flow cytometric analyses. Red lines refer to negative controls. Blue lines stand for the OASC group. (E) Endothelial-to-mesenchymal transition (EMT) of HCECs (cultivated in BM) in passage 6 (P6) under phase-contrast microscopy. (F) The morphology of CM-HCECs in passage 6 (P6), and passage 9 (P9) under phase-contrast microscopy. Scale bar: 100 μm.

**Additional file 2: Figure S2. **Clinical observation of the control group and the TR2Y group after transplantation of CM-HCECs. Slit-lamp photographs show the monkey corneal endothelial dysfunction model in the control group (left). Slit-lamp photographs show the monkey corneal endothelial dysfunction model following injection of CM-HCECs in the TR2Y group (monkey 2, right). Images were obtained at day 7, day 14, day 21, 1 month, and 2 months after surgery.

## Data Availability

The RNA-seq data was deposited to NCBI SRA and the BioProject No. is PRJNA796842. The datasets used and/or analyzed during the current study are available from the corresponding author on reasonable request.

## Abbreviations

BM: Basal culture medium
CCK-8: Cell counting Kit-8
CCT: Central corneal thickness
CECs: Corneal endothelial cells
CM: Conditioned medium
CM-HCECs: Cultivated HCECs with conditioned medium
DEGs: Differential expression genes
DM: Descemet’s membranes
DMEK: Descemet’s membrane endothelial keratoplasty
DMEM-LG: Dulbecco’s modified Eagle’s medium low glucose
DSAEK: Descemet's stripping automated endothelial keratoplasty
DSEK: Descemet’s stripping endothelial keratoplasty
ECM: Extracellular matrix
EMT: Endothelial-to-mesenchymal transition
FECD: Fuchs endothelial corneal dystrophies
HCECs: Human corneal endothelial cells
H&E: Hematoxylin and eosin
ICE: Iridocorneal endothelial syndrome
IOP: Intraocular pressure
KP: Keratic precipitates
MAPK: Mitogen-activated protein kinase
OASCs: Orbital adipose-derived stem cells
OASC-CM: Conditioned medium obtained from orbital adipose-derived stem cells
PBS: Phosphate-buffered saline
PK: Penetrating keratoplasty
SKPs: Skin-derived precursors
TR2Y: Transplantation after 2 years
ZO-1: Zonula occludens-1
