# Supplementary figures and images for: Long-term observation after transplantation of cultured human corneal endothelial cells for corneal endothelial dysfunction
Source: Stem Cell Res Ther. 2022 Jun 3;13:228. doi: 10.1186/s13287-022-02889-x (PMC9166479; doi:10.1186/s13287-022-02889-x)

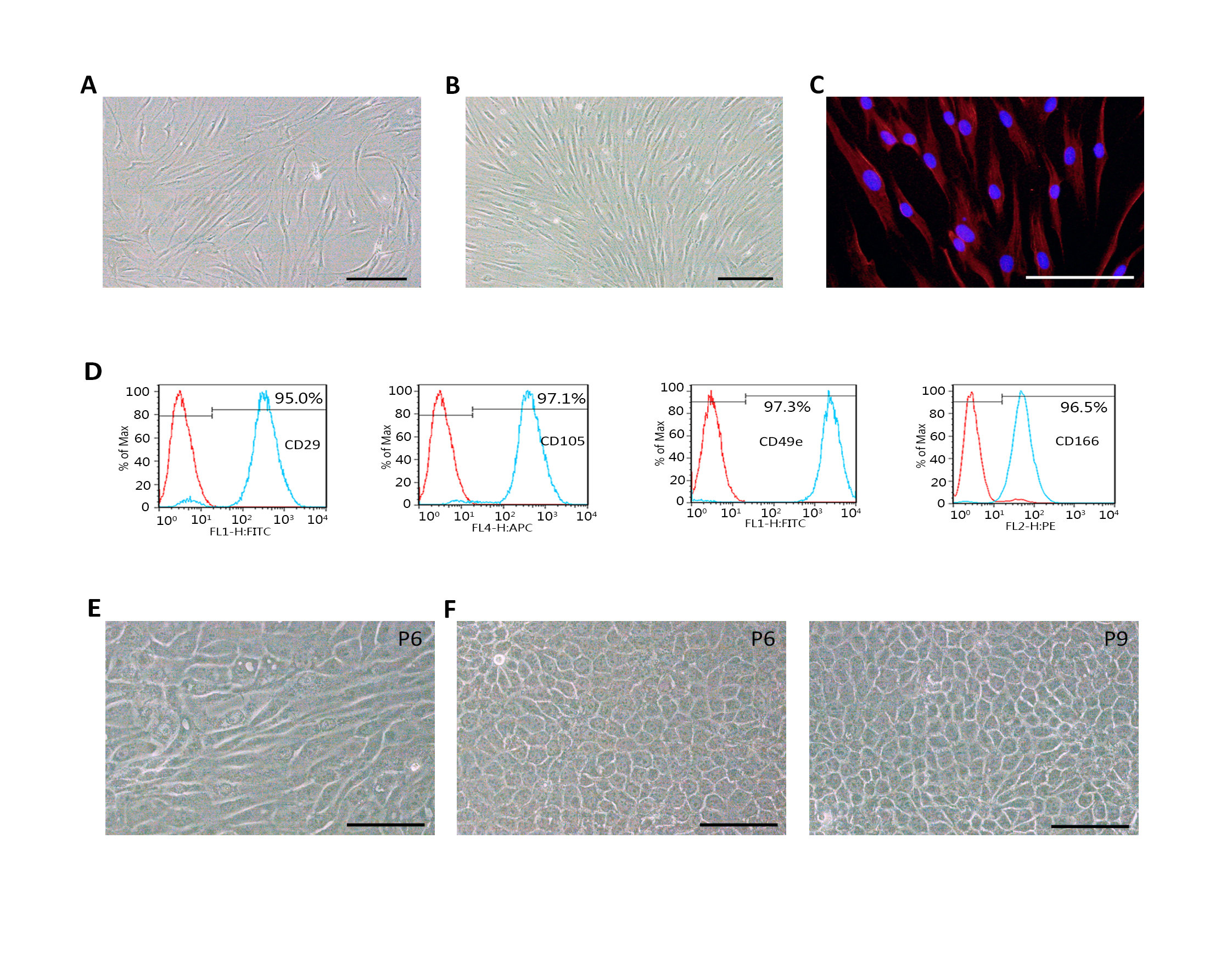

Supplement: Supplementary file 1 — Additional file 1: Figure S1. Cell culture, and characteristics of human orbital adipose-derived stem cells (OASCs), HCECs (cultivated in BM), and CM-HCECs. (A), (B) OASCs were adherent, spindle-shaped, fibroblast-like cells under phase-contrast microscopy. (C) Expression of vimentin as determined by immunofluorescence. (D) Expression of related cell markers determined by flow cytometric analyses. Red lines refer to negative controls. Blue lines stand for the OASC group. (E) Endothelial-to-mesenchymal transition (EMT) of HCECs (cultivated in BM) in passage 6 (P6) under phase-contrast microscopy. (F) The morphology of CM-HCECs in passage 6 (P6), and passage 9 (P9) under phase-contrast microscopy. Scale bar: 100 μm. [file 13287_2022_2889_MOESM1_ESM.tif]

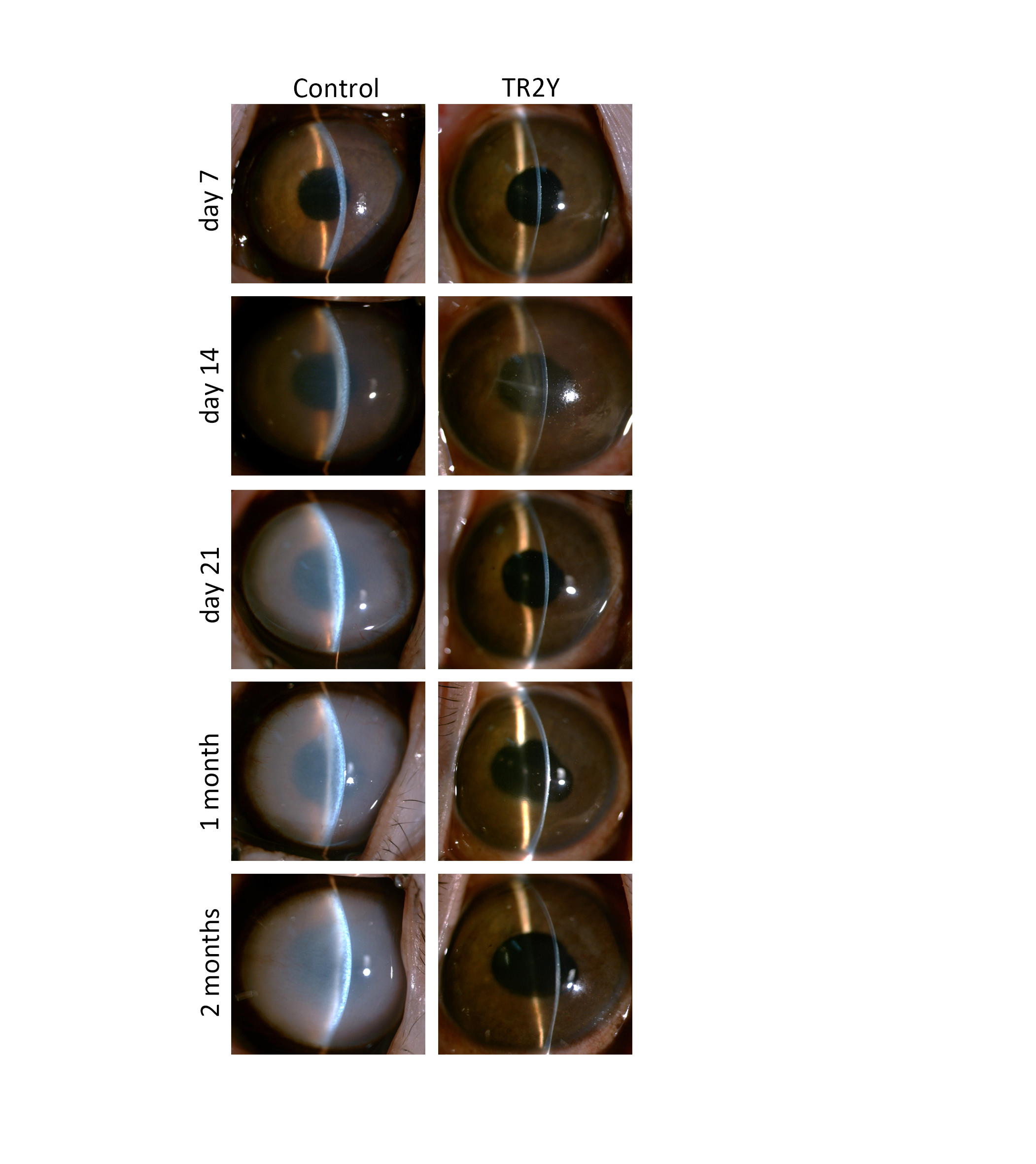

Supplement: Supplementary file 2 — Additional file 2: Figure S2. Clinical observation of the control group and the TR2Y group after transplantation of CM-HCECs. Slit-lamp photographs show the monkey corneal endothelial dysfunction model in the control group (left). Slit-lamp photographs show the monkey corneal endothelial dysfunction model following injection of CM-HCECs in the TR2Y group (monkey 2, right). Images were obtained at day 7, day 14, day 21, 1 month, and 2 months after surgery. [file 13287_2022_2889_MOESM2_ESM.tif]
